# Plasma donor-derived cell-free DNA kinetics after kidney transplantation using a single tube multiplex PCR assay

**DOI:** 10.1371/journal.pone.0208207

**Published:** 2018-12-06

**Authors:** Els M. Gielis, Charlie Beirnaert, Amélie Dendooven, Pieter Meysman, Kris Laukens, Joachim De Schrijver, Steven Van Laecke, Wim Van Biesen, Marie-Paule Emonds, Benedicte Y. De Winter, Jean-Louis Bosmans, Jurgen Del Favero, Daniel Abramowicz, Kristien J. Ledeganck

**Affiliations:** 1 Laboratory of Experimental Medicine and Pediatrics, University of Antwerp, Antwerp, Belgium; 2 Biomedical Informatics Research Network Antwerp (Biomina), University of Antwerp/Antwerp University Hospital, Antwerp, Belgium; 3 Advanced Database Research and Modelling (ADReM), Department of Mathematics and Computer Science, University of Antwerp, Antwerp, Belgium; 4 Department of Pathology, Antwerp University Hospital, Antwerp, Belgium; 5 Multiplicom N.V., part of Agilent Technologies, Niel, Belgium; 6 Renal Division, Ghent University Hospital, Ghent, Belgium; 7 Histocompatibility and Immunogenetic Laboratory, Belgian Red Cross Flanders, Mechelen, Belgium; 8 Department of Nephrology and Hypertension, Antwerp University Hospital, Antwerp, Belgium; University of Liège, BELGIUM

## Abstract

**Background:**

After transplantation, cell-free DNA derived from the donor organ (ddcfDNA) can be detected in the recipient’s circulation. We aimed to quantify ddcfDNA levels in plasma of kidney transplant recipients thereby investigating the kinetics of this biomarker after transplantation and determining biological variables that influence ddcfDNA kinetics in stable and non-stable patients.

**Materials and methods:**

From 107 kidney transplant recipients, plasma samples were collected longitudinally after transplantation (day 1–3 months) within a multicenter set-up. Cell-free DNA from the donor was quantified in plasma as a fraction of the total cell-free DNA by next generation sequencing using a targeted, multiplex PCR-based method for the analysis of single nucleotide polymorphisms. A subgroup of stable renal transplant recipients was identified to determine a ddcfDNA threshold value.

**Results:**

In stable transplant recipients, plasma ddcfDNA% decreased to a mean (SD) ddcfDNA% of 0.46% (± 0.21%) which was reached 9.85 (± 5.6) days after transplantation. A ddcfDNA threshold value of 0.88% (mean + 2SD) was determined in kidney transplant recipients. Recipients that did not reach this threshold ddcfDNA value within 10 days after transplantation showed a higher ddcfDNA% on the first day after transplantation and demonstrated a higher individual baseline ddcfDNA%.

**Conclusion:**

In conclusion, plasma ddcfDNA fractions decreased exponentially within 10 days after transplantation to a ddcfDNA threshold value of 0.88% or less. To investigate the role of ddcfDNA for rejection monitoring of the graft, future research is needed to determine causes of ddcfDNA% increases above this threshold value.

## Introduction

After transplantation, cell-free DNA derived from the donor organ (ddcfDNA) can be detected in the recipient’s circulation. Its release might be associated with cell damage in the graft thereby indicating a role of ddcfDNA as a biomarker for graft injury after transplantation[1]. Based on prospective data in heart and liver transplant recipients, it is known that a high amount of ddcfDNA is released in the circulation of the transplant recipient in the immediate post-engraftment phase, followed by swift decreases in the plasma fractions of ddcfDNA (proportion of circulating cell-free DNA that is donor-derived) approximately one week after transplantation[2, 3]. In heart, lung and liver transplant recipients with an acute rejection of the graft, increased plasma ddcfDNA% were observed compared to recipients with a stable graft function thereby suggesting a role for ddcfDNA as a biomarker of rejection[2–4]. However, kinetics of the ddcfDNA are less studied after kidney transplantation, as longitudinal data from kidney transplant recipients are currently lacking. Hence, a reference ddcfDNA% in kidney transplant recipients is still unknown.

In addition, limitations of several methods for ddcfDNA quantification impede clinical implementation of these assays. While some ddcfDNA quantification techniques based on amplification of chromosome Y associated genes are only suitable in gender-mismatched transplantation settings[5, 6], other universal techniques based on whole genome shotgun sequencing to quantify donor specific single nucleotide polymorphisms (SNPs) are time consuming, require a complex bio-informatical analysis and long turnaround times[2, 7]. Apart from sequencing of SNPs, Beck *et al*. introduced a ddcfDNA quantification technique based on the amplification of 41 SNPs using digital droplet PCR technology[8].

The aim of this study was to quantify ddcfDNA in plasma of kidney transplant recipient using a single tube multiplex polymerase chain reaction (PCR)-based assay for the amplification of SNPs (Sequido, Multiplicom (part of Agilent Technologies)). Therefore, we designed a prospective, observational multicenter study in which ddcfDNA was quantified in serial plasma samples of kidney transplant recipients. In this manuscript, we investigated the normal kinetics of this biomarker in stable kidney graft recipients in the early post-transplantation period. In addition, we aimed to determine a plasma ddcfDNA threshold value in stable kidney transplant recipients.

## Materials and methods

### Sample collection

To investigate ddcfDNA kinetics in kidney transplant recipients, adult patients who received a kidney transplantation were enrolled in a multicenter, prospective, observational clinical study at the Antwerp University Hospital and the Ghent University Hospital between October 2014 and March 2017 after providing a written informed consent. At the Antwerp University Hospital, all consecutive patients who received a kidney transplantation were enrolled, except for patients with a history of non-kidney transplantation, multi-organ transplant recipients and patients who preferred not to participate in the study. In addition to these exclusion criteria, patients that were referred from general hospitals were not included at the University Hospital of Ghent to ensure a complete follow-up of three months.

After renal transplantation, blood samples were collected in Cell-Free DNA BCT collecting tubes (Streck, Nebraska, U.S.) at 10 time points: day 1, day 3, week 1, week 2, week 3, week 4, week 6, week 8, week 10 and month 3. Additional blood samples were collected the day from a protocol biopsy procedure and during hospital admission for a rise in serum creatinine and/or the performance of an indication biopsy. From each recipient, a whole blood EDTA sample was collected before transplantation to isolate genomic DNA (gDNA). From every deceased donor, gDNA was provided by the HILA (Histocompatibility and Immunogenetic Laboratory, Belgian Red Cross Flanders, Mechelen, Belgium) and from every living donor, a whole blood EDTA sample or buccal swab (Isohelix, Kent, U.K) was collected after a written informed consent to isolate gDNA.

For the determination of the inter- and intra-assay variation of the ddcfDNA quantification assay, blood from two healthy donors was collected in BCT collecting tubes (Streck, Nebraska, U.S). From each donor, one EDTA tube was collected for gDNA isolation.

This study was approved by the Ethics committees of the Antwerp University Hospital (file number 14/30/308) and the Ghent University Hospital (file number 2014–1200). All blood samples were collected after a written informed consent.

### Cell-free (cfDNA) and genomic DNA extraction

Within 2 days after collection, blood samples were centrifuged following a 2-step centrifugation protocol (1600g for 10 min and 3200g for 20 min at room temperature) to remove the cells. Within 6 months of storage at -80°C, plasma samples were thawed at room temperature and cfDNA was extracted from a maximal amount of 5 ml plasma using the QIAamp circulating nucleic acid kit (Qiagen, Venlo, The Netherlands) and a Vac elut SPS-24 manifold (Agilent Technologies, California, U.S) to provide vacuum pressure. All reagent volumes were adjusted to the starting volume of the plasma. Other cfDNA extraction steps were performed according to the manufacturer’s protocol. Genomic DNA was extracted from EDTA blood samples or buccal swabs by a standard salting-out procedure[9] or using a Buccalyse DNA extraction kit (Isohelix), respectively.

### Quantification of donor-derived cell-free DNA

#### Sequido workflow

**Fig 1:**
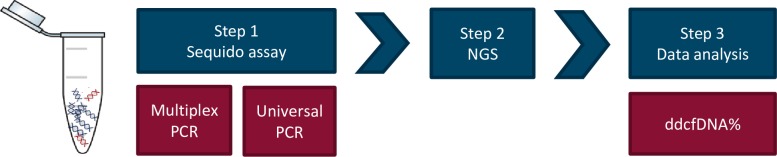
Sequido workflow. Step 1: Multiplex PCR amplification of 1027 SNP containing amplicons followed by a universal PCR step adding platform-specific sequencing adapters and sample molecular identifiers (MID) after which the library is ready for sequencing. Step 2: Next generation sequencing on an Illumina MiSeq or Illumina NextSeq 500/550 instrument. Step 3: Processing of generated sequencing data resulting in a ddcfDNA% for each sample. PCR: polymerase chain reaction, NGS: next generation sequencing, ddcfDNA: donor-derived cell-free DNA.

For the purpose of this study, Multiplicom (part of Agilent Technologies) designed the Sequido, which workflow is schematically presented in Fig 1. The Sequido assay is a single tube, highly multiplexed PCR-based assay amplifying targeted regions of the human genome. The assay workflow consists of two steps. Within a first step, 1027 Single nucleotide polymorphisms (SNPs) located on autosomal chromosomes (average population frequency of 20.75%) are amplified in a multiplex PCR (amplicons size ranges from 65–85 base pairs (bp)). Next generation sequencing (NGS) platform-specific sequencing adapters and sample molecular identifiers are added in a second universal PCR step, after which the library is ready for sequencing. The Sequido assay was designed, optimized and manufactured at Multiplicom (a part of Agilent Technologies) following a standard commercial manufacturing process.

All samples (both the donor/recipient gDNA and cfDNA samples) were processed with the Sequido assay and subsequently sequenced according the manufacturer’s instructions for use. Samples were sequenced on an Illumina MiSeq instrument using v3 chemistry kit or on an Illumina NextSeq 500/550 instrument using Mid output v2 kit or High output v2 kit. A maximum of 16 samples were pooled on MiSeq, 86 samples on NextSeq Mid output and 192 samples on NextSeq High output. The sequencing read length was always 1x76bp.

The generated Sequido sequencing data were processed with the Sequido Reporter, a private-cloud based analysis platform hosted in Amazon Web Services (AWS) (Amazon, Frankfurt) developed by Multiplicom (Agilent Technologies) adhering to the highest safety standards. For each patient, a ‘sample group’, each containing the identifiers of a single donor gDNA sample, a single recipient gDNA sample and multiple longitudinal cfDNA samples, was defined in the Sequido Reporter and FASTQ files were manually uploaded and linked to the appropriate sample identifiers. Analysis was performed on each sample group.

In the first step of the analysis, a sample group was converted to a set of trios: donor gDNA, recipient gDNA and 1 cfDNA sample (1 trio for each cfDNA follow-up time point).

Using a competitive mapping approach, the SNP status (number of wild type reads, number of variant reads, the ratio of variant over sum of variant and wild type, total coverage) of each amplicon was defined in each of the three samples. Amplicons with a low coverage were removed from the analysis. The recipient and the donor SNP allele constitution at all 1027 SNP positions was determined based on the observed variant allele frequency respectively in the recipient and the donor genomic DNA sample. Hereto, a variant frequency below 5% indicated a homozygous wild type allele SNP constitution, a variant frequency between 35% and 65% pointed to a heterozygous SNP constitution, and a variant allele frequency exceeding 95% indicated a homozygous variant SNP constitution. If the variant allele frequencies in the gDNA samples deviated from these expected ranges, the amplicon was rejected in the sample. On average > 95% of the amplicons were retained for further processing.

**Fig 2:**
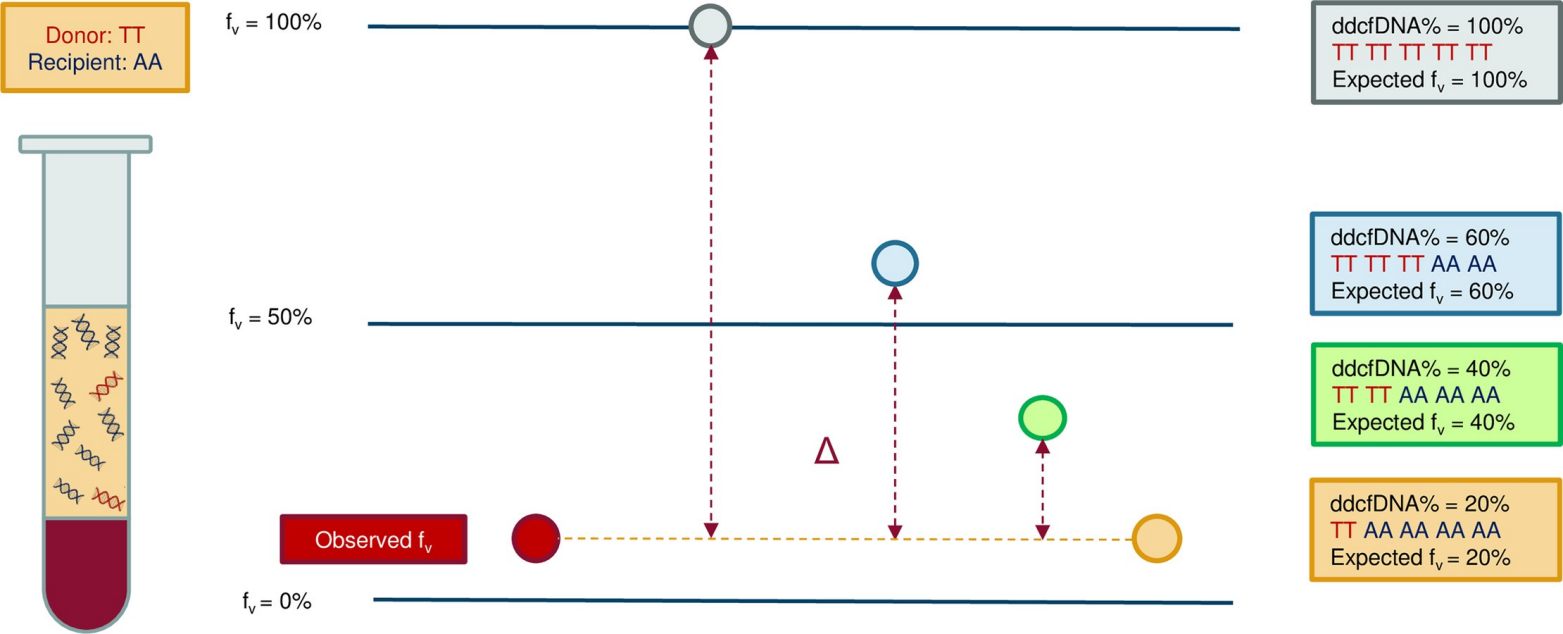
Principle of the Sequido assay. Differences in the expected and observed variant allele frequencies (f_v_) at a SNP position for different donor cell-free DNA percentages in a sample. For this certain SNP, the donor has a homozygous constitution for the variant allele (TT) and the recipient is homozygous for the wild type allele (AA). The expected variant allele frequencies (f_v_) are depicted for a ddcfDNA% of 100% (grey circle), 60% (blue circle), 40% (green circle) and 20% (orange circle). The expected variant allele frequency for every ddcfDNA% mixture is visualized in the frameworks with identical colors, according to the recipient (AA) and donor (TT) allele constitution in this example. Δ: difference in the expected and observed variant allele frequencies for a certain ddcfDNA%; f_v_: variant allele frequency; A: wild type allele; T: variant allele; ddcfDNA: donor-derived cell-free DNA.

Given that both the donor and acceptor allele status are known for each SNP, an expected variant allele frequency for each SNP could be calculated for a certain donor fraction in a cfDNA sample which contains a mixture of recipient and donor cfDNA fragments. For each SNP position, the difference between the expected and the observed allele frequency should be as low as possible. An example is depicted in Fig 2.

A maximum-likelihood like approach was used to estimate the most probable donor fraction thereby selecting the donor fraction for which the difference of the expected and observed variant allele frequencies were as low as possible for all SNPs. An error score was calculated for different theoretical donor fractions. The lowest error score indicates the most probable donor fraction (ddcfDNA%) in the sample. The following error score model was used (f_v_: variant allele frequency):

$$error\;score=\sum_{i=1}^{1027}{\left(SNP\;{fv}_{i}^{expected}(f)-SNP\;{fv}_{i}^{observed}\right)}^{2}$$

#### Assay precision of the Sequido assay

For the determination of the precision of the ddcfDNA quantification assay, cfDNA mixtures were created. Hereto, cfDNA extracts from each healthy blood donor were pooled and cfDNA concentration was measured using the QubitTM HS dsDNA Assay Kit (Thermo Fisher Scientific). A ddcfDNA% mixture of 15% was prepared by spiking cfDNA from the first donor into cfDNA of the second donor (the recipient). Other mixtures of 10%, 5%, 1% and 0.5% ddcfDNA% were obtained by further diluting the 15% mixture sample with cfDNA volumes of the recipient.

Multiplex PCR assays were performed for each created cfDNA mixture (3 assays for the 15%, 10%, 5% and 0.5% ddcfDNA mixtures and 6 assays for the 1% ddcfDNA mixture). Following each multiplex assay, a universal PCR and subsequent sequencing were performed in duplicate.

For the 15%, 10%, 5%, 1% and 0.5% ddcfDNA mixtures, the inter-assay coefficient of variation (CV) (based on the ddcfDNA% of different multiplex PCRs) were respectively 0.48%, 1.37%, 0.42%, 5.24%, and 9.08%. An intra-assay variability was calculated based on the universal PCR and sequencing duplicates thereby showing an intra-assay CV range (min-max) of 0.18% - 1.27% (15% ddcfDNA% mixture), 0.22% - 0.76% (10% ddcfDNA% mixture), 0.01–1.77% (5% ddcfDNA% mixture), 1.94–7.62% (1% ddcfDNA% mixture) and 0.62–18.93% (0.5% ddcfDNA% mixture).

### Clinical data collection and biopsy evaluation

For clinical data collection, an electronic case report form was created using the online software platform edc2go (Genae, Antwerp, Belgium). In the current analysis, the following recorded post-transplantation events were analyzed: delayed graft function (the need of dialysis within the first week after transplantation[10]), treated urinary tract infections, surgical adverse events requiring treatment (hematoma, hemorrhagic shock or other complications), episodes of hydronephrosis requiring intervention (placement of JJ stent or pyelostomy) and pre-renal acute kidney injury with recovery after fluid treatment.

All protocol and indication biopsies were evaluated centrally and blinded from the results of the ddcfDNA measurements by a single experienced nephropathologist (A. Dendooven, M.D., PhD) at the Antwerp University Hospital according to the Banff 2013 classification of allograft rejection[11, 12].

### Selection of stable kidney transplant recipients

In this study, we aimed to investigate the ddcfDNA kinetics and establish a plasma ddcfDNA threshold value in stable kidney graft recipients. Hereto, we selected a subgroup of stable kidney transplant recipients from the entire study cohort after all patients finished the study follow-up of three months after transplantation. The stable graft group was selected based on the following criteria: absence of acute kidney injury (i.e. no serum creatinine increase of ≥ 0.3 mg/dl compared to the previous study follow-up visit according to the KDIGO guidelines[10]) and the absence of ddcfDNA peaks (ddcfDNA% > 1%) within the study follow-up of three months. The presence of an untreated borderline rejection episode on protocol biopsy was not considered as an exclusion criterion for the stable recipient subgroup.

### Statistical analysis

#### Description of ddcfDNA kinetics after transplantation

Within a subgroup of stable transplant recipients, natural cubic splines were used to fit ddcfDNA% curves.

#### Variables that influence Day 1 ddcfDNA%

An elastic net regression was used to create a model to predict the ddcfDNA% on day 1 based on recipient, donor, transplantation and day 1 clinical characteristics, removing those recipients with incomplete data. The associated p-value was calculated by training the model in a 10-fold cross validation setting by comparing the predicted values on the held-out data to the true values.

#### Calculation of a ddcfDNA threshold value

To determine a threshold ddcfDNA% in stable transplant recipients, multiple linear models were fitted to every patient. Each model contained an intercept and slope coefficient and was fitted on three consecutive measurement points: the first model included measurements 1 to 3, the second 2 to 4, etc. The models with a slope coefficient less than 0.05 were considered stabilized with the first time point included in these stabilized models considered as the first day of stabilization. The mean of all stabilized measurements of all stable transplant recipients was calculated to determine a threshold ddcfDNA value.

As a next step in the statistical analysis, we investigated the observed ddcfDNA kinetics and patient’s individual baseline levels of the entire study cohort. We intended to identify clinical adverse events that are associated with abnormal ddcfDNA kinetics in the immediate post-transplantation phase and clinical variables that correlate with individual baseline ddcfDNA%.

#### Variables that influence individual baseline ddcfDNA%

In the entire study cohort of transplant recipients, individual median baseline ddcfDNA% were determined for each recipient thereby taking the median of all stabilized measurements (below the baseline ddcfDNA% established in the group of stable transplant recipients). A Spearman correlation analysis or ANOVA was performed to determine the correlation between clinical parameters and patient’s individual baseline ddcfDNA levels, depending on the type of parameter. All univariate test p-values were adjusted with a strict Bonferroni correction for multiple testing; reported p-values are always the adjusted values and p-values below 0.05 were considered as statistical significant.

An unpaired Student’s t-test was used to test for differences in median baseline ddcfDNA% and the ddcfDNA% on day 1 between recipients that stabilized before day 10 and recipients that did not.

#### Variables that influence immediate ddcfDNA kinetics

Abnormal ddcfDNA kinetics in the early post-transplantation period were established by fitting two models to each patient’s data (exponential decay or sigmoidal decay) according to the following formulas (sp: switch point):

$$ddcfDNA\%∼N_{0}\;\exp\;\left(-b*day\right)$$

$$ddcfDNA\%∼N_{0}\frac{1}{1+\exp\;\left(day-sp\right)}$$

Corresponding to the median ddcfDNA% kinetic of stable transplant recipients, an exponential decay was used as a reference kinetic. The parameters of each model were fitted with nonlinear least squares. For each fitted model, the Akaike Information Criterium (AIC) was calculated. This AIC value quantified the quality of the fit, taking into account the number of parameters, and allowed for comparing different models. Lower AIC values indicated a superior better model. The patients that exhibited a lower AIC value for the sigmoidal fit were determined to be patients with abnormal ddcfDNA kinetics. Association between abnormal kinetics and clinical events in the unstable post-transplantation phase were investigated using a Chi-square or Fisher’s Exact test.

All analyses were performed in R (v3.2.4). Boxplots were created using IBM SPSS Statistics (v.24).

## Results

### Patients and samples

In total, 107 patients were enrolled into this study. Of these recipients, 1036 plasma samples were collected at 10 (84 recipients), 9 (16 recipients) and 8 (5 recipients) follow-up visits. In 2 recipients, plasma sampling was ended after six visits because of a transplant nephrectomy. Samples on day 1 were collected within 12 hours after transplantation (29.7%), between 13–24 hours (44.6%) or after 24 hours (15.8%). The time interval of sampling after transplantation was not recorded for 10 (9.9%) of the day 1 samples. Protocol biopsies were performed as planned in 57 recipients between week 10 and 3 months. A blood sample concomitant with the protocol biopsy was available for 43 patients. Supplementary blood samples (total number of 46) were drawn from 28 transplant recipients hospitalized for acute graft dysfunction and/or indication biopsy.

### Kinetics of the ddcfDNA% in stable kidney transplant recipients

**Fig 3:**
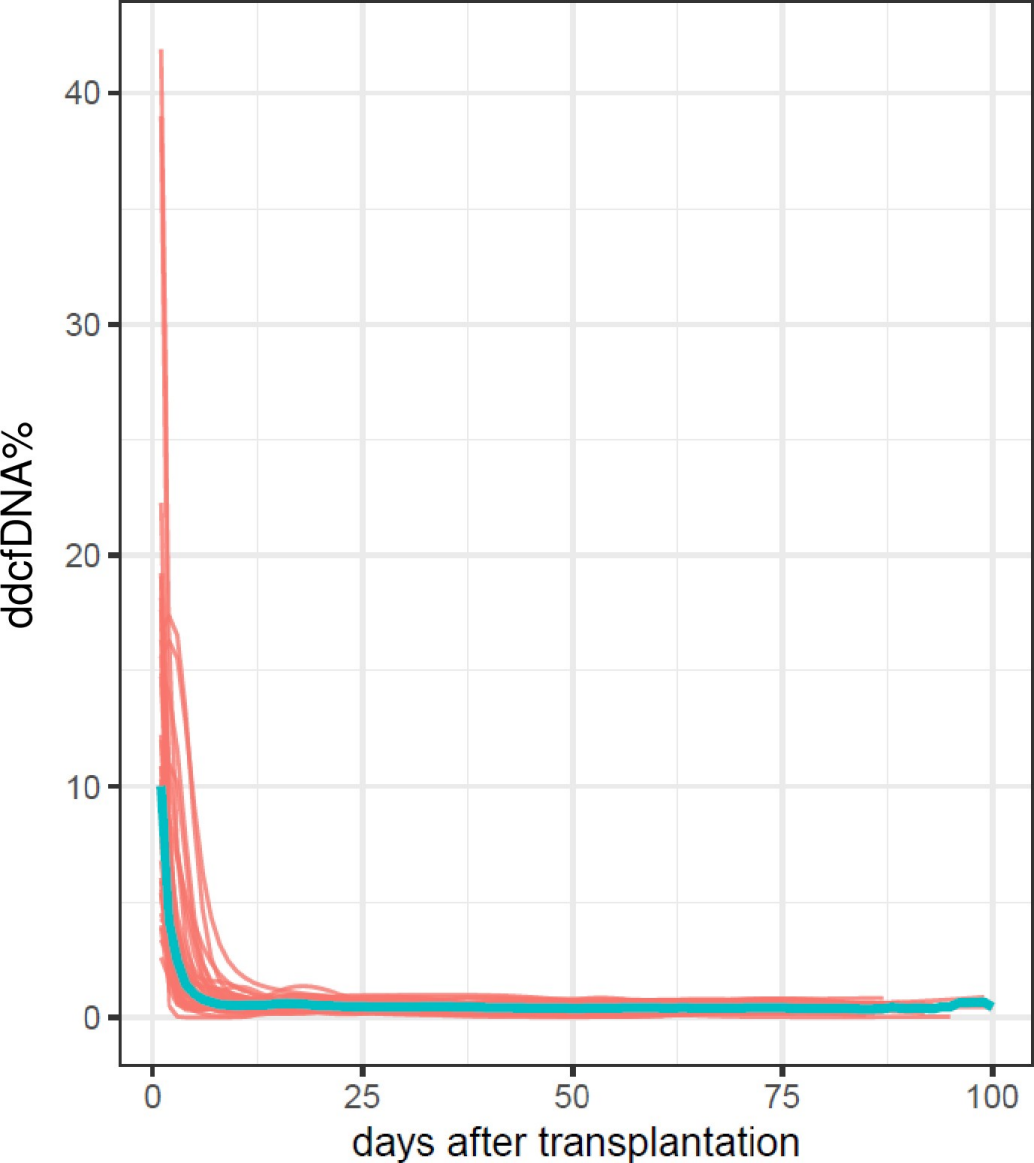
Fit ddcfDNA% curves of kidney transplant recipients. Fit ddcfDNA% curves from 42 stable kidney transplant recipients are shown in red. The median ddcfDNA% fit curve is shown in blue. ddcfDNA: donor-derived cell-free DNA.

Fourty-two patients (39.3%) were identified as stable kidney transplant recipients, whose clinical characteristics are provided in Table 1. In Fig 3, ddcfDNA% fit curves of these recipients are shown. At the first day after transplantation, median ddcfDNA fractions of 10.02% were measured, ranging from 2.60% to 41.89%. Afterwards, fractions of ddcfDNA decreased with an exponential kinetic to a mean (SD) value of 0.46% (0.21%) that was reached on average 9.85 ± 5.6 days after transplantation. We calculated the threshold ddcfDNA value as the mean + 2SD—thus 0.46% + 2(0.21%)—resulting in a ddcfDNA% of 0.88%. An elastic net regression model used for the prediction of the ddcfDNA% on day 1 (p = 0.055) revealed that a younger recipient age, higher second warm ischemia times, and better kidney function (eGFR CKD-EPI) on day 1 resulted in higher ddcfDNA%, while a male recipient gender and transplantation with a living donor were negatively associated with the ddcfDNA% on day 1. The time of blood collection after transplantation was a significant negative predictor, with a lower ddcfDNA% when the blood sample was collected at a later time point after transplantation (>24 hours).

**Table 1:** Recipient, donor and transplantation characteristics of the subgroup of stable kidney transplant recipients.

Number of recipients	n = 42
Center 1 (n (%))	36 (85.7)
Center 2 (n (%))	6 (14.3)
Recipient characteristics (at transplantation):	
Male gender (n, (%))	30 (71.4)
Age (years)	52 (18–69)
PRA (%)	0 (0–97)
Residual diuresis (n, (%))	
0 ml/day	9 (21.4)
≤ 500 ml/day	8 (19.0)
> 500 ml/day	21 (50.0)
Not known	4 (9.5)
Donor characteristics:	
Donor Type (n, (%))	
Living donor	7 (16.7)
Deceased donor	35 (83.3)
Donor age (years)	45.4 (± 11.2)
Donor-recipient compatibility characteristics:	
Number of HLA mismatches (A, B, DR) (n, (%))	
0	2 (4.8)
1	1 (2.4)
2	13 (31.0)
3	18 (42.9)
4	7 (16.7)
5	0 (0)
6	1 (2.4)
Transplantation characteristics:	
Ischemia times	
Cold ischemia time (hours)	10.9 (± 6.5)
2nd warm ischemia time (minutes)	29.6 (± 8.8)
Maintenance immunosuppressive treatment:	
Prednisolone (n, (%))	42 (100.0)
MMF/ Azathioprin/ Everolimus (n, (%))	39 (92.8) / 1 (2.4) / 2 (4.8)
Tacrolimus/ Cyclosporin (n, (%))	34 (81.0) / 8 (19.0)
Day 1:	
Number of samples (n, (%))	39 (92.9)
eGFR (CKD-EPI) (ml/min)	12.0 (4.0–39.0)
Time interval sample collection after transplantation (n, (%))	
(≤12 hours)	14 (35.9)
(13–24 hours)	18 (46.2)
(>24 hours)	6 (15.4)
Not known	1 (2.6)
Baseline ddcfDNA%:	
Mean (SD)	0.46% (0.21)
Median (IQR)	0.45% (0.26)

n: number; PRA: panel reactive antibodies; HLA: Human leukocyte antigen; eGFR CKD-EPI: estimated Glomerular Filtration Rate using the Chronic Kidney Disease Epidemiology collaboration equation. Normally distributed continuous data are presented as a mean (± standard deviation, SD), skewed data are presented as median (min-max).

### Kinetics of the ddcfDNA% in the entire cohort of patients

#### Kinetics of ddcfDNA% in the early post-transplantation period

The kinetics of the ddcfDNA during the early post-transplantation phase were investigated for all patients, thereby including 312 samples collected before day 10 after transplantation as this day was identified as the average day of ddcfDNA% stabilization. During this early post-transplantation phase, a higher urine protein to creatinine ratio and a sampling closer to the transplantation date correlated positively with the ddcfDNA% in a multivariate analysis, while no correlations with either kidney function (serum creatinine (r = -0.044; p = 1.00), eGFR (r = 0.088; p = 0.16) and diuresis), markers of inflammation (C-reactive protein (CRP), neutrophils), or other vital parameters (temperature, blood pressure) were observed.

**Fig 4:**
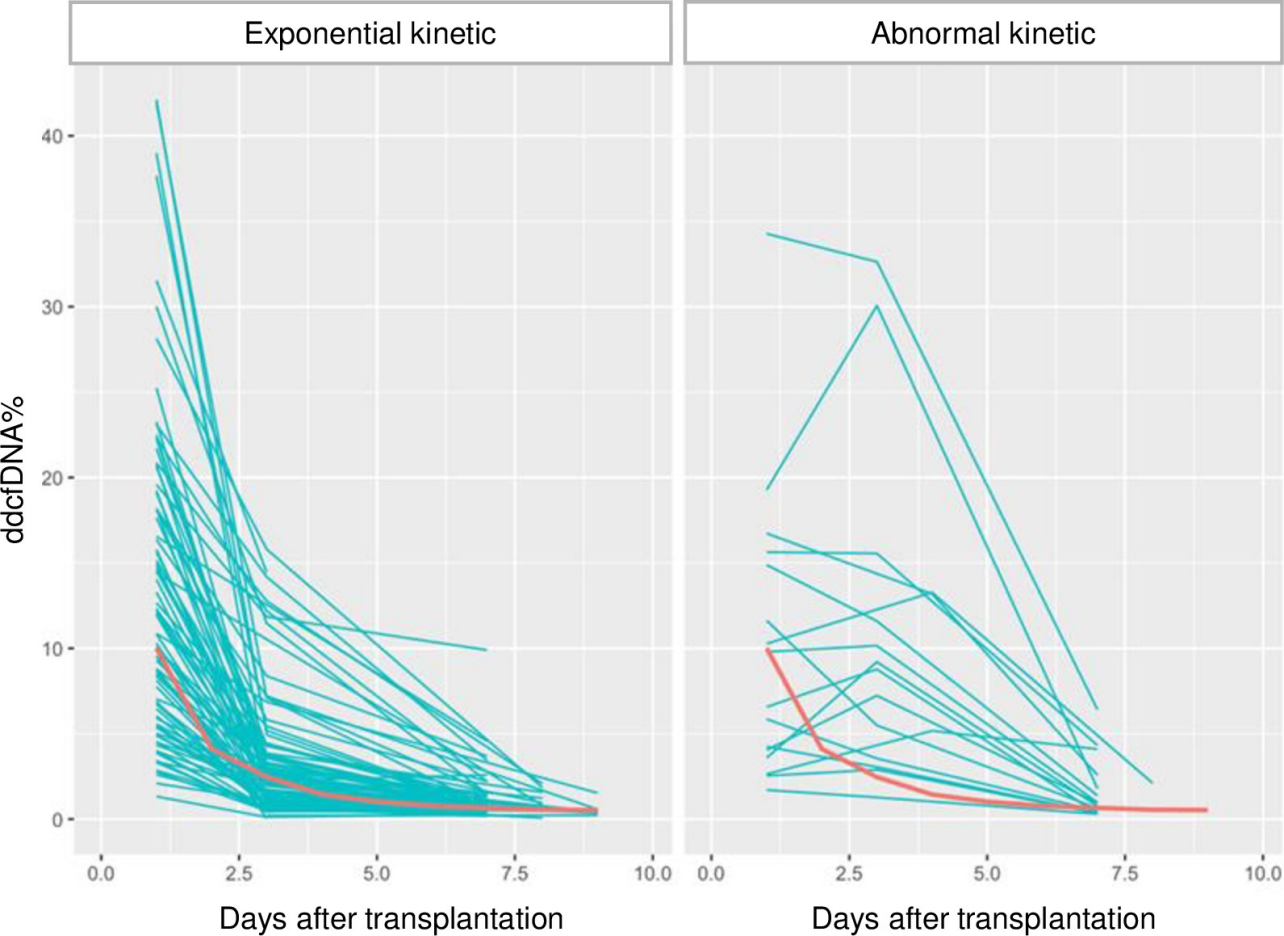
Fit ddcfDNA% curves from all kidney transplant recipients. Fit ddcfDNA% curves from all kidney transplant recipients (exponential kinetic: n = 90; abnormal kinetic: n = 16) during the unstable post-transplantation phase before day 10. One recipient was excluded because of a lack of sufficient samples to define the ddcfDNA% kinetic. The reference median fit curve of the subgroup of stable renal transplant recipients is shown in red in both graphs. ddcfDNA: donor-derived cell-free DNA.

Sixteen patients showed an abnormal non-exponential decline in ddcfDNA% within 10 days after transplantation (Fig 4). No association was observed between an abnormal ddccfDNA% kinetic and the occurrence of delayed graft function or the performance of an (early) indication biopsy (p = 0.696). However, the occurrence of one or more other early adverse event including a urinary tract infection, pre-renal acute kidney injury, surgical complication or an episode of hydronephrosis was significantly associated with an abnormal ddcfDNA decline after transplantation (p = 0.001).

#### Individual baseline ddcfDNA values in the entire cohort

**Fig 5:**
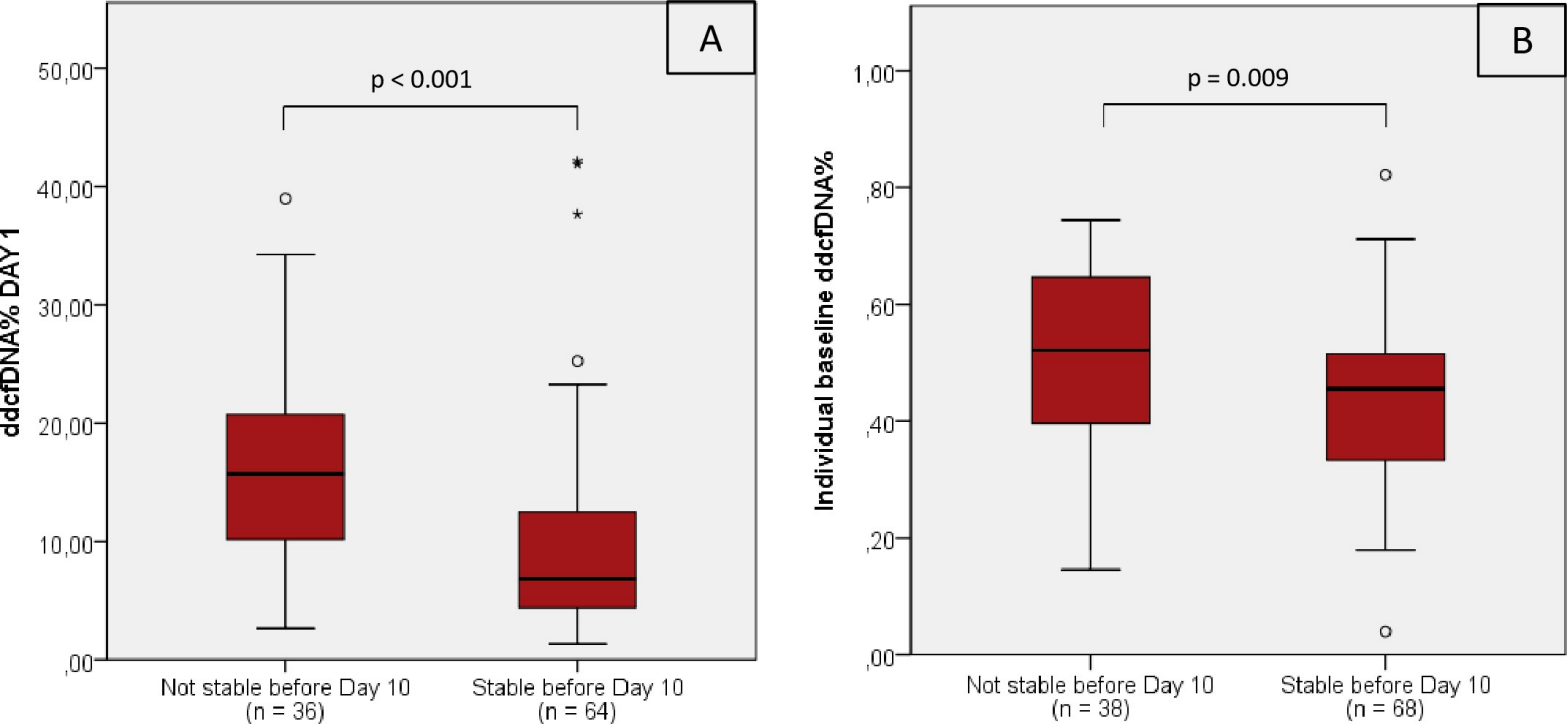
ddcfDNA% at day 1 and individual baseline ddcfDNA%. Differences in ddcfDNA% at day 1 (A) and median baseline ddcfDNA% (B) between recipients who reached a ddcfDNA% below the threshold value of 0.88% before day 10 and recipients who did not reach the threshold value by that time. Individual median baseline ddcfDNA% were determined for each recipient thereby taking the median of all stabilized measurements (below the ddcfDNA threshold value established in the group of stable transplant recipients). One recipient never reached a ddcfDNA% below 0.88%. Plasma samples from the first day after transplantation were not available from 6 recipients (n = 2 not stable before day 10; n = 4 stable before day 10). p < 0.05 with a Student’s t-test. ddcfDNA: donor-derived cell-free DNA; n = number of recipients.

The individual’s median baseline ddcfDNA values varied below the ddcfDNA threshold value with a mean (±SD) plasma ddcfDNA fraction of 0.47% (± 0.15%). As shown in Table 2, the individual baseline values did not correlate with recipient, donor or transplantation characteristics. Only a trend towards a positive correlation was observed for the recipient PRA (panel reactive antibodies) before transplantation (r = 0.282; p = 0.078). Interestingly, patients who did not reach a ddcfDNA% below the threshold value of 0.88% at day 10 (n = 38) exhibited a higher individual baseline ddcfDNA% (0.52% (± 0.14%) vs. 0.44% (± 0.14%); p = 0.009) and also started from a higher ddcfDNA% at the first day after transplantation (16.62% (± 7.94%) vs. 10.21% (± 8.86%); p < 0.001) compared to recipients who decreased below the 0.88% before day 10 (Fig 5).

**Table 2:** Univariate correlation analysis of median ddcfDNA baseline values of the entire study cohort with recipient, donor or transplantation characteristics.

Recipient characteristics (at transplantation)	Correlation coefficient	Adjusted p-value
Study Center	N/A	1.00
Gender	N/A	1.00
Age	-0.057	1.00
BMI	-0.135	1.00
Residual diuresis (0 ml/day, ≤500 ml/day, >500 ml/day)	N/A	1.00
Primary renal disease (glomerular/chronic interstitial nephritis/cystic disease/renal vascular disease/diabetes/other)	N/A	1.00
PRA (%)	0.282	0.078
Presence donor specific antibodies	N/A	1.00
Donor kidney in situ from previous transplantation	N/A	1.00
Diabetes Mellitus Type II	N/A	1.00
Current smoking	N/A	1.00
Obesitas (BMI ≥ 30 kg/m)	N/A	1.00
Chronic inflammatory diseases	N/A	1.00
Donor and transplantation characteristics	Correlation coefficient	Adjusted p-value
Age	0.004	1.00
Type (living/deceased)	N/A	1.00
BMI	0.127	1.00
BMI Difference recipient-donor	-0.174	1.00
Number of HLA mismatches in HLA A and B	-0.086	1.00
Number of HLA mismatches in HLA DR	-0.143	1.00
Cold ischemia time	0.088	1.00
2^nd^ warm ischemia time	0.137	1.00
Induction therapy (IL2-RA, ATG)	N/A	1.00

BMI: Body mass index; PRA: panel reactive antibodies; HLA: Human leukocyte antigen; IL2-RA: Interleukin-2 receptor antagonist; ATG: Anti-thymocyte globulin; N/A: not applicable. Spearman correlation coefficients are shown for continuous variables. Categorical variables were tested using an analysis of variance (ANOVA) to test difference between the different groups (only p-value reported). p-values are adjusted according to a Bonferroni correction for multiple testing.

## Discussion

This study was designed to investigate the kinetics of donor-derived cell-free DNA after transplantation thereby determining a plasma ddcfDNA threshold value in kidney transplant recipients. We showed that after transplantation, plasma ddcfDNA% decreased to a mean ddcfDNA% of 0.46% (± 0.21%) approximately 10 days after transplantation in stable graft recipients. This time interval of ddcfDNA% stabilization was also observed in heart and liver transplant recipients[2, 3]. A ddcfDNA threshold value of 0.88% (mean + 2SD) was withheld in kidney transplant recipients. Kidney transplant recipients that were not stabilized within 10 days after transplantation, showed a higher ddcfDNA% on the first day after transplantation and remained on a higher individual baseline ddcfDNA% throughout the post-transplantation course of three months.

In this study, we used a novel assay for the quantification of ddcfDNA% in plasma samples of renal transplant recipients based on targeted multiplex amplification of 1027 highly polymorphic SNPs. Technical validation of this assay demonstrated a good assay reproducibility with an increase in assay precision at higher ddcfDNA%. This universal ddcfDNA quantification approach can be used in every donor and recipient pair, which makes it attractive for clinical use. Snyder *et al*. introduced a whole genome sequencing approach, thereby analyzing about 50.000 SNPs [7], which renders this technique expensive and dependent on complex bio-informatical analyzes. Recently, Grskovic and coworkers introduced an assay based on the targeted amplification of a set of 266 SNPs[13]. In that assay, donor DNA is not implemented making it possible to measure ddcfDNA fractions in the absence of donor material. However, as a consequence, the upper limit of detection is 25% ddcfDNA% and a formula needs to be applied to adjust for donor relatedness. Using digital droplet PCR technology, Beck *et al*. introduced a ddcfDNA quantification method based on the analysis of 41 preselected SNPs[8]. However, on average, only a set of 3 SNPs allowed discrimination between donor and recipient DNA (informative SNP) and could be used for ddcfDNA quantification. For digital PCR, quantitative precision improves with increasing number of PCR analyses performed. Therefore, several thousand digital PCRs need to be performed, requiring the use of automated platforms. Such automated platforms using microfluidics are available but are expensive.

We observed high levels of ddcfDNA% (10%) in the circulation of the renal recipient on the first day after transplantation which is in accordance with previous findings on the first day after heart (4%)[2], lung (26%)[4] and liver transplantation (70%)[3], thereby possibly reflecting ischemia-reperfusion injury in the graft related to the transplant process. According to our analysis in stable renal transplant recipients, transplantation with a living donor kidney resulted in a lower ddcfDNA% on the first day, which is in line with previous observations in kidney transplant recipients[14]. In addition, we observed higher ddcfDNA% on the first day after transplantation in younger and female recipients and after a transplantation with a longer warm ischemia time.

In stable renal transplant recipients, a mean ddcfDNA% of 0.46% (± 0.21%) was reached approximately 10 days after transplantation. In line with our data, recently, Bromberg *et al*. reported a mean plasma ddcfDNA% of 0.34% (± 0.58%) in 93 stable kidney transplant recipients thereby including at least 3 plasma ddcfDNA measurements of each recipient collected from 1 month up to 12 months after transplantation[15]. In heart transplant recipients, mean fractions of 0.06% were measured in rejection-free samples[2], while ddcfDNA% below 10% indicated graft integrity in liver transplant recipients[3]. Tissue mapping of plasma cfDNA of healthy, non-transplant recipients revealed that besides hematopoietic cells, the liver is a major contributor to the circulating cfDNA pool [16].

In our study, the differences observed in individual’s baseline values were not related to recipient, donor nor transplantation characteristics, although it is known from the literature that several conditions, including obesity and chronic inflammatory disorders are associated with an increased total circulating cfDNA[17, 18], thereby possibly influencing the ddcfDNA fraction.

In kidney transplant recipients, it has to be questioned whether increased levels of ddcfDNA are related to impaired kidney function rather than reflecting actual graft damage. It is currently unknown which mechanisms are involved in the clearance of cfDNA from the plasma. A study on the clearance of fetal DNA from the maternal circulation showed that only 0.2–19% of the fetal DNA is cleared by the kidney indicating that renal excretion is a minor route for the clearance of circulating fetal (non-host) DNA[19]. Furthermore, no differences in total cfDNA concentrations were observed in patients with chronic kidney disease (CKD) compared to healthy controls, nor between patients with different stages of CKD[20–22]. In the present study, ddcfDNA was measured as a fraction of the total circulating cfDNA, not by quantification of absolute ddcfDNA levels. We did not find a significant correlation between plasma ddcfDNA fractions and the kidney function of the recipient (serum creatinine: r = -0.044, p = 1.00; eGFR: r = 0.088, p = 0.16). On the other hand, the amount of plasma ddcfDNA% correlated with the presence of proteinuria in the early post-transplant period. Our data thus indicate an association of ddcfDNA release with graft injury rather than with renal function.

The important strength of this study is the longitudinal set-up in which kidney transplant recipients were sampled from day 1 until 3 months after transplantation, resulting in improved insights in ddcfDNA kinetics necessary to analyse and interpret further studies properly. There are also limitations of this study. We limited our study follow-up to 3 months, which is in contrast with other studies in heart, lung and liver transplantation, investigating ddcfDNA until 1 to 2 years after transplantation[2–4]. In our study, we identified 42 recipients as stable transplant recipients. Of these patients, only a limited number of recipients received a kidney from a living donor, which could explain the observation that we could not find a difference in the ddcfDNA baseline value in recipients with different donor types. Finally, using this new technique, results should be validated in an independent cohort of kidney transplant recipients.

In conclusion, after kidney transplantation, plasma ddcfDNA% decrease with an exponential kinetic to a ddcfDNA threshold value of 0.88% within approximately 10 days after transplantation. It still has to be questioned whether this biomarker could have a potential role in kidney allograft monitoring. To investigate the diagnostic capacity of ddcfDNA as a marker for acute rejection, further analyses are needed in which causes of increases of the ddcfDNA% above the threshold value are evaluated.
